# Supplementary figures and images for: A forward genetic screen identifies Dolk as a regulator of startle magnitude through the potassium channel subunit Kv1.1
Source: PLoS Genet. 2021 Jun 1;17(6):e1008943. doi: 10.1371/journal.pgen.1008943 (PMC8195410; doi:10.1371/journal.pgen.1008943)

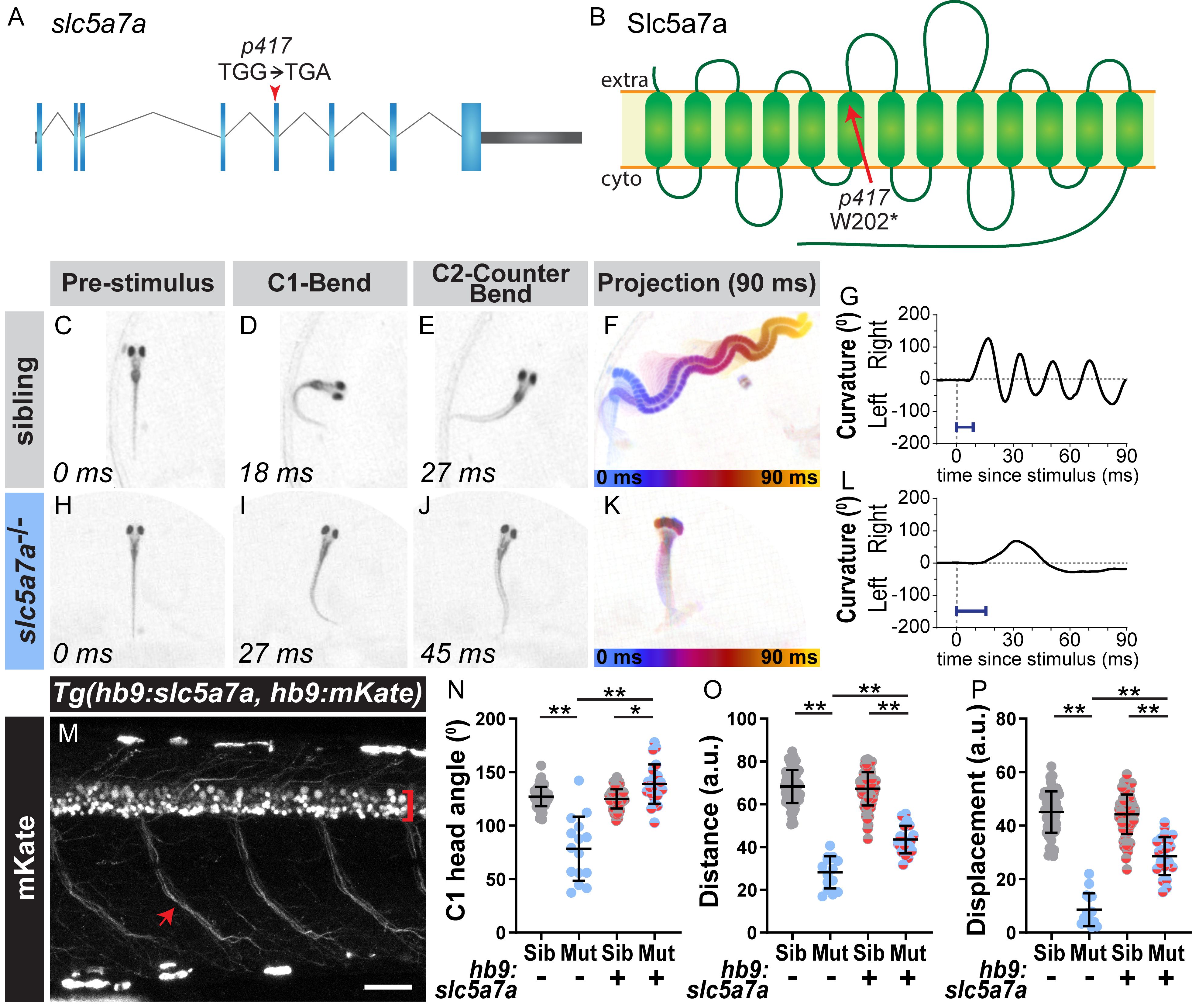

Supplement: S1 Fig — (A) Gene structure for slc5a7a, with the nonsense mutation from the screen (allele p417) noted. (B) Protein structure for Slc5a7a on the plasma membrane, with the predicted amino acid change from the screen mutation indicated. In contrast to siblings (C-G), slc5a7a mutants (H-L) display a weak startle response, with reduced bend and counter bend angles, as well as uncoordinated movement. Blue bar = latency in G,L. (M) slc5a7a-/-; Tg(hb9;slc5a7a, hb9:mKate) larvae express mKate and slc5a7a in motor neurons. mKate is expressed from a second hb9 promoter to allow visualization of expressing neurons without disrupting protein function. Bracket indicates motor column and arrow indicates motor nerve axons. Scale bar = 50 μM. (N-P) Kinematic parameters of the acoustic startle response in slc5a7a siblings and mutants with or without hb9:slc5a7a transgene. Each point represents an average of ten trials for an individual fish. n≥15 larvae, *p = 0.002, **p<0.0001 (one-way ANOVA with Tukey correction for multiple comparisons). (TIF) [file pgen.1008943.s002.tif]

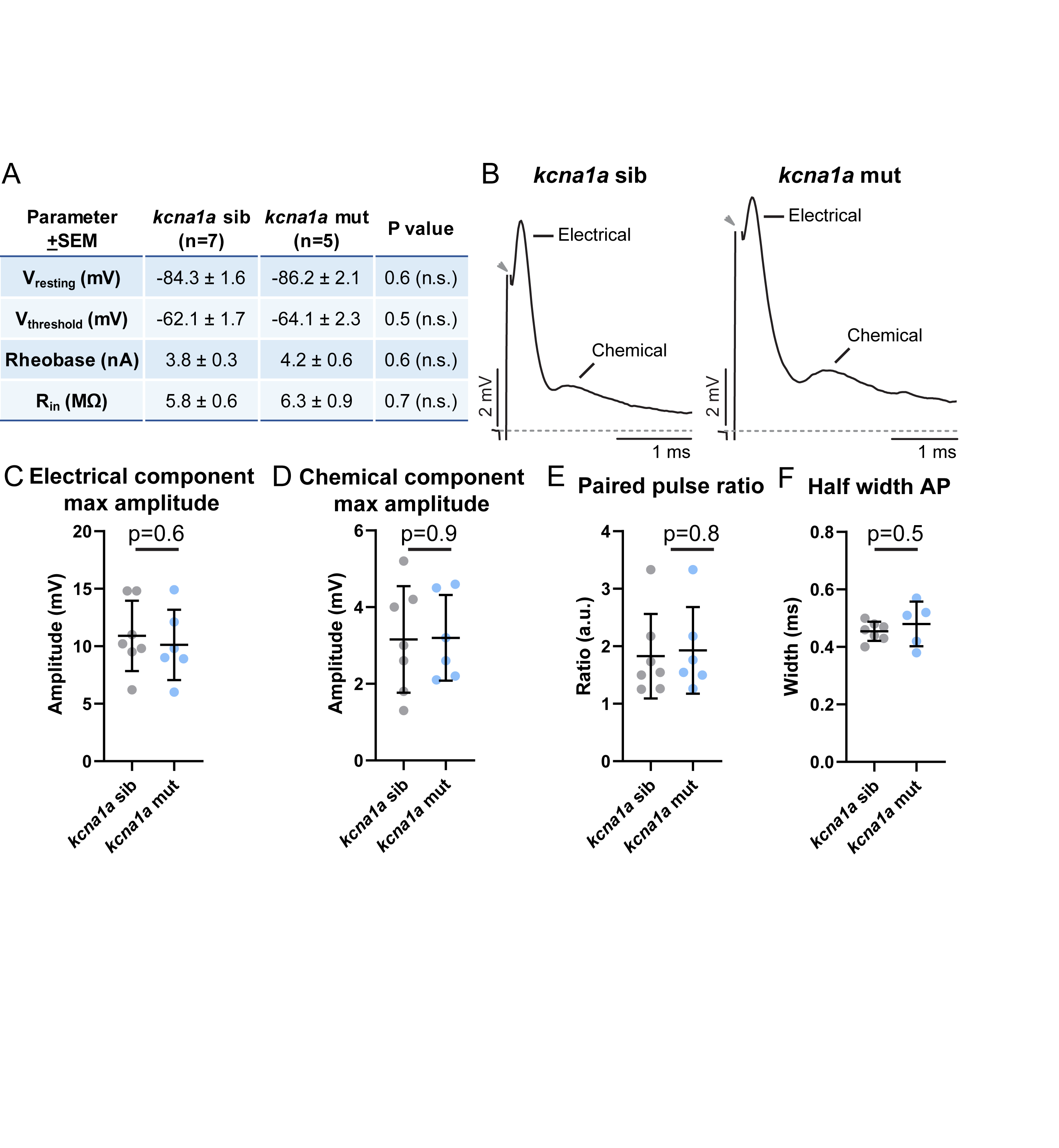

Supplement: S2 Fig — (A) Averaged measurements of Vresting, Vthreshold, Rheobase, and Rin in kcna1a sibling and mutant larvae. n.s. = not significant. (B) Representative mixed synaptic responses on the Mauthner cell evoked by electrical stimulation of auditory afferents (club endings) in kcna1a sibling (left) and mutant (right) larvae (responses represent the average of at least 10 single traces). The maximal amplitude of the electrical component (C), chemical component (D), paired-pulse ratio of the chemical component (E), and half width AP (F) of the mixed synaptic response in kcna1a sibling or mutant Mauthner cells are not significantly different. n = 7 kcna1a sibling larvae, n = 6 (C-E)/5 (F) kcna1a mutant larvae. Mann-Whitney tests used for p-values. (TIF) [file pgen.1008943.s003.tif]
